# Beta cortical oscillatory activities and their relationship to postural control in a standing balance demanding test: influence of aging

**DOI:** 10.3389/fnagi.2023.1126002

**Published:** 2023-05-05

**Authors:** Yang Hu, Steven J. Petruzzello, Manuel E. Hernandez

**Affiliations:** ^1^Department of Kinesiology and Community Health, College of Applied Health Sciences, University of Illinois at Urbana-Champaign, Urbana, IL, United States; ^2^Department of Kinesiology, College of Health and Human Science, San José State University, San Jose, CA, United States; ^3^Department of Biomedical and Translational Sciences, Carle Illinois College of Medicine, University of Illinois at Urbana-Champaign, Urbana, IL, United States

**Keywords:** sensory perturbations, mechanical perturbations, aging, cortical control of posture, EEG

## Abstract

**Background:**

Age-related changes in the cortical control of standing balance may provide a modifiable mechanism underlying falls in older adults. Thus, this study examined the cortical response to sensory and mechanical perturbations in older adults while standing and examined the relationship between cortical activation and postural control.

**Methods:**

A cohort of community dwelling young (18–30 years, *N* = 10) and older adults (65–85 years, *N* = 11) performed the sensory organization test (SOT), motor control test (MCT), and adaptation test (ADT) while high-density electroencephalography (EEG) and center of pressure (COP) data were recorded in this cross-sectional study. Linear mixed models examined cohort differences for cortical activities, using relative beta power, and postural control performance, while Spearman correlations were used to investigate the relationship between relative beta power and COP indices in each test.

**Results:**

Under sensory manipulation, older adults demonstrated significantly higher relative beta power at all postural control-related cortical areas (*p* < 0.01), while under rapid mechanical perturbations, older adults demonstrated significantly higher relative beta power at central areas (*p* < 0.05). As task difficulty increased, young adults had increased relative beta band power while older adults demonstrated decreased relative beta power (*p* < 0.01). During sensory manipulation with mild mechanical perturbations, specifically in eyes open conditions, higher relative beta power at the parietal area in young adults was associated with worse postural control performance (*p* < 0.001). Under rapid mechanical perturbations, specifically in novel conditions, higher relative beta power at the central area in older adults was associated with longer movement latency (*p* < 0.05). However, poor reliability measures of cortical activity assessments were found during MCT and ADT, which limits the ability to interpret the reported results.

**Discussion:**

Cortical areas are increasingly recruited to maintain upright postural control, even though cortical resources may be limited, in older adults. Considering the limitation regarding mechanical perturbation reliability, future studies should include a larger number of repeated mechanical perturbation trials.

## 1. Introduction

With a rapidly increasing number of individuals over 65 years of age globally, more people face functional impairment associated with the aging process, such as reductions in balance function and increases in fall risk (Lord et al., 2018). Nearly 30% of older adults report falling (Bergen et al., 2016), and falling once doubles the chances of falling again (O’loughlin et al., 1993). Falls lead to injuries, reductions in quality of life, and even death(Kannus et al., 1999; Hartholt et al., 2011; Bergen et al., 2016), with death rates in the United States having increased 30% from 2007 to 2016 for older adults (Burns and Kakara, 2018). Considering the severe consequences of falls in older adults, it is essential to further our understanding on the mechanisms underlying falls and identification of modifiable factors that can reduce the fall rate in older adults.

Standing postural control ability tends to decline with increased age, and is significantly associated with falls (Quijoux et al., 2020). Postural control is defined as the act of maintaining, achieving or restoring a state of balance that may involve either a fixed-support or a change in support response (Pollock et al., 2000). Studies investigating postural control commonly utilize posturography to quantitatively assess postural stability (Sullivan et al., 2009). Specifically, larger postural sway areas have been associated with worse postural stability and a higher risk of falling (Johansson et al., 2017). Older adults have demonstrated increases in postural sway ranges and center of pressure (COP) velocities while standing with eyes open or close (Roman-Liu, 2018). Older adults have also exhibited larger COP peak displacements after perturbations while standing in comparison to young adults (Kanekar and Aruin, 2014; Quijoux et al., 2020).

Upright postural and balance control requires a complex interplay within and between the sensory and the motor systems. Furthermore, there is strong evidence for the crucial contribution of the cerebral cortex in the control of balance (Jacobs and Horak, 2007; Maki and McIlroy, 2007; Papegaaij et al., 2014). A growing number of studies have demonstrated increasing cortical activities in more challenging balance conditions (Wittenberg et al., 2017; Malcolm et al., 2021; Barollo et al., 2022; Tsai et al., 2022). Specifically, cortical activity and high-order cognitive processes are important when static postural control is challenged by mechanical and sensory perturbations, as the responsive adjustments depend on the integration of reliable sensory feedback and planning and execution of appropriate motor responses (O’Connor and Kuo, 2009; O’Connor et al., 2012; Francis et al., 2015; Franz et al., 2015, 2017; Goodworth et al., 2015; Malcolm et al., 2021; Tsai et al., 2022).

As suggested by current electroencephalography (EEG) studies, multiple brain regions and cortical beta band (13–30 hz) electrical activities are involved in maintaining upright static balance in adults (Ibitoye et al., 2021; Malcolm et al., 2021; Barollo et al., 2022; Tsai et al., 2022). The parietal-occipital region, frontal-central region, and occipital lobe are involved in response to visual challenges while standing (Chang et al., 2016; Malcolm et al., 2021; Tsai et al., 2022). Parietal and central areas beta band power were sensitive to proprioceptive challenges while standing (Tse et al., 2013). Electrical activity at the central coronal reference curve, such as Cz (related to sensory and motor cortex), Pz (related to parietal lobe), Fz (related to frontal lobe), and nearby electrodes are associated with responses to mechanical perturbations while standing (Adkin et al., 2006; Jacobs et al., 2008; Mochizuki et al., 2009; Smith et al., 2014). Previous work also investigated the association between cortical activities and postural control abilities while standing. Specifically, in response to backward mechanical perturbation, higher cortical beta powers are associated with larger perturbations (Ghosn et al., 2020).

Recent EEG work (Ibitoye et al., 2021; Malcolm et al., 2021; Tsai et al., 2022) has started to examine age-related changes on the cortical control of upright stance, confirming prior evidence suggesting (Rubega et al., 2021) that the cortical neural activities during the balance task also changes along with aging and are associated with poor postural control and higher fall risk (St George et al., 2021). However, while modifications to stance and visual feedback have been primarily used in studies examining age-related changes, a wider examination of age-related changes due to sensory manipulation and mechanical perturbations could provide valuable information about aging’s effect on cortical contributions to balance control in more complex environments, crucial for linking to changes in fall risk.

Thus, the purpose of this study was to examine (a) the effect of aging on the cortical response to sensory manipulation and mechanical perturbations while standing and (b) the relationship between cortical activation and the underlying postural control. We hypothesized that (1) compared to young adults (YA), older adults (OA) would demonstrate significantly higher relative beta power at postural control-related cortical areas, specifically at Fz, Cz, and Pz; and (2) increased relative beta band power would be found as task difficulty increased, particularly in OA. Secondarily, we examined the association between relative beta power and postural control performance.

## 2. Methods

This study consisted of a single session cross-sectional experimental design. Community-dwelling adults with the following inclusion criteria were recruited (1) Right-handed; (2) Young adults between 18 to 30 years of age and older adults over 65 years of age. (3) Free of chronic or acute neurological conditions, such as Parkinson’s disease, Huntington’s disease, stroke, epilepsy, and seizures; and (4) Free of severe heart conditions, such as heart attack, heart failure, and angina. Exclusion criteria included: (1) Cognitive impairment, as defined by a Modified Telephone Interview for Cognitive Status (TICS-M) questionnaire score lower than 18 (Cook et al., 2009); (2) Physical disability or inability to walk independently without an assistive device; and (3) Severe chronic pain that limits physical function. Once in the study, all participants read and signed a written informed consent form. The protocol and procedures have been reviewed and approved by the Institutional Review Board of the University of Illinois Urbana Champaign.

To incorporate sensory and mechanical perturbation and provide comparable results to previous studies, the Sensory Organization Test (SOT), Motor Control Test (MCT), and Adaptation Test (ADT) were used in this study. Participants were asked to stand as still as possible in all three tests, while high-density electroencephalography (EEG), and center of pressure (COP) data was recorded. SOT, MCT, and ADT are clinically used standardized instrumented balance tests performed using the SMART EquiTest-Clinical Research System (SECRS, Neurocom, a division of Natus). The SOT is designed to assess a patient’s use of sensory systems that contribute to balance and identify any abnormalities in the systems (Mcguirk, 2005). The six conditions of the SOT manipulate or eliminate information normally delivered to the patient’s eye, head, feet, and joints. Specifically, there are three trials per condition and 20 s per trial in the SOT. The SOT measures an individual’s ability to suppress the misleading information from the conflicting senses and use the remaining sensory input to maintain an upright stance (Honaker and Criter, 2013). Thus, in this study, the SOT introduces visual and somatosensory perturbations using sway-referenced mechanical ankle rotations, as part of the different sensory and minor mechanical perturbations presented to participants. To provide higher levels and two different types of mechanical perturbation, the MCT and ADT were conducted after the SOT. The MCT contains six conditions, including three forward and three backward translations graded in magnitude [small (2.8 degrees/s), medium (6.0 degrees/s), and large (8.0 degrees/s)], which were scaled to subject’s height, with three trials of each condition and 2.5 s per trial (Jacobs and Horak, 2007; NeuroCom International, 2008). The ADT consists of two different conditions (toes-up, toes-down with an 8-degree platform rotation at a rate of 20 degrees/s) with five trials of each condition and 2.5 s per trial. In each trial, a sudden and randomly timed movement (8 degree over 400 ms) of the platform about the ankle in the toes-up (dorsiflexion) and toes-down (plantar flexion) planes elicit an automatic balance response (NeuroCom International, 2008) to participants. The MCT contains six conditions, including three forward and three backward translations graded in magnitude [small (2.8 degrees/s), medium (6.0 degrees/s), and large (8.0 degrees/s)], which were scaled to each subject’s height, with three trials of each condition and 2.5 s per trial (Jacobs and Horak, 2007; NeuroCom International, 2008). Furthermore, baseline functional balance, cognitive, and psychological function was evaluated to help control for potential covariates in cortical activation and postural control. Functional balance was evaluated by the MiniBESTest battery. The repeatable battery for the assessment of neuropsychological status (RBANS) was also used to identify and characterizing abnormal cognitive decline (Randolph et al., 1998) of the participants. Lastly, the fall risk of the participants was assessed by the Falls Efficacy Scale-International (FES-I; Delbaere et al., 2010).

### 2.1. Cortical activation assessment

High-density EEG data from a 64-channel active system (ActiCHamp system, Brain Vision LLC, Morrisville, NC USA) were recorded at 1 kHz, using the average of the left and right mastoids as reference. EEG sensor placement was based on the international 10–10 system. All three tests were recorded as one continuous EEG recording. Raw EEG data were imported into EEGLAB (version 2020.0) using MATLAB (The MathWorks, Natick, MA, USA) for pre-processing. Pre-processed data were then labeled based on the start and end markers of each trial under each condition in each test, and epoch to eliminate preparation and resting time in between each trial. Thus, 20s epochs from SOT paradigms and 2.5 s epochs from MCT and ADT paradigms were used for followed EEG analysis. As supported by previous literature regarding the aging effect on cortical control of postural, the main outcome measurement of the EEG data was relative beta (13–30 Hz) power (% Power) at Fz, Cz, and Pz (Adkin et al., 2006; Jacobs et al., 2008; Mochizuki et al., 2009; Tse et al., 2013; Smith et al., 2014; Chang et al., 2016; Ghosn et al., 2020). Equation 1 was used to calculate relative beta power for each participant in each unique condition. In which, power was computed by ‘bandpower’ function in MATLAB. This function computes the average power in the input signal vector based on the selected frequency range. The total power was calculated to ½ sampling rate to provide reliable results. Thus, the total power was calculated with a range of 0 to 500 Hz, while band of interest is 13-30 Hz for the beta wave. Relative beta power was calculated at the electrode level; thus, the results were specific to electrodes and bands of interest.


(1) $$\begin{array}{l} Relativepowerofbandofinterest=\\ \frac{absolutepowerofthebandofinterest}{totalpowerofthecondition} \end{array}$$

Before calculating Relative beta power, a grand average calculation was performed on each unique condition, thus eliminating the trial effect. Additionally, an interclass correlation coefficient (ICC) analysis were used to determine the trial effect use the epoch data before grand average. Each condition’s clean EEG data was re-referenced to a subject level baseline average voltage; therefore, the results describe the changes relevant to a baseline condition (eyes open standing).

### 2.2. Postural control assessment

COP data were collected through the SERCS. The primary outcome measure from the COP data in SOT is Equilibrium Score. Equilibrium score reflecting the overall coordination under each SOT condition and calculated by comparing the angular differences between the patient’s estimated maximum and minimum sagittal plane body sway to a theoretical maximum displacement (12.5 degree) and provided a score between 100 (no body sway) to 0 (fall; Honaker and Criter, 2013). The major outcome measure in MCT is the time elapsed (Latency) which SECRS directly reports. Latency is defined as the time in milliseconds between the onset of a translation and the onset of the patient’s active force response to the induced sway. Specifically, latency detection is based on differentiation of force plate data from each foot. The resulting velocities are analyzed with four separate algorithms, each of which produces a latency estimate. Latency estimates that differ by 10 milliseconds or less are taken as identical. The longest latency estimate is then considered the latency. The number of algorithms that find the same latency is the “quality factor,” or degree of consistency. A quality factor of 4 indicates all four algorithms agree. When no two algorithms agree, a quality factor of 1 is assigned, and the longest latency estimate is used. If none of the algorithms detect a onset of response, no latency can be identified, and a quality of 0 appears on the display/printout. Essentially, this determines how long it takes to go from the onset of the perturbation to the onset of the center of gravity balance correction response to maintain upright stance, with shorter latency corresponding to a faster reaction to the perturbation (NeuroCom International, 2008; Shepard and Janky, 2008). The primary outcome measure from the COP data in ADT is the sway energy score (range 0 to 300) directly reported by SECRS. This score was calculated based on COP position in the anterior–posterior direction during each perturbation condition (Vanicek et al., 2013) using the following formula:

Sway Energy = C1* PY′(RMS) + C2* PY′′ (RMS).

Where PY′ denote velocity and PY′′ denote acceleration, C1 and C2 are weighting constants used to give dimensionless energy values:

$C1=\frac{1}{in/\sec}$ and $C2=\frac{0.025}{\sec^{2}}$

A higher sway energy score corresponds to a higher force required to overcome the postural instability (Trueblood et al., 2018).

### 2.3. Statistical analysis

All the statistical analyses were performed using R (R 4.0.3, Rstudio 1.2.1335). There were four sets of statistical analyses that were performed to answer the research questions. The independent t-test was used to test for cohort demographic differences. The ICC analysis was used to assess the trial effect in each test paradigm. For primary outcome measurements, linear mixed effect models (LMMs) were used to identify the cohort differences for cortical activities and postural control performance. Specifically, LMMs of relative power of beta band at Fz, Cz, and Pz were used to test the hypotheses of aging effect and age-task interaction effects on relative power during SOT, MCT, and ADT. LMMs were also constructed on COP equilibrium score, COP average latency score, and COP sway energy to identify the aging effect on postural control from the biomechanical aspect. When significant interaction effects were found, Least Square Means (LSM) posthoc comparisons were performed, and a *p* < 0.05 was considered statistically significant. Moreover, Spearman correlations were used to investigate the relationship between relative beta power and COP indices in each test, and between relative beta power in each test and miniBest score. The correlation strength was evaluated based on Evans’s method (Evans, 1996). Thus, the relative beta power was averaged on test conditions level and on subject level accordingly. Additional details of LMMs are described in Supplementary materials Section 1.2.

## 3. Results

### 3.1. Descriptive characteristics

Overall, the two participant groups were not significantly different in cognitive function, gender, and self-reported fall risks (Table 1). In comparison to the young adult (YA) group, the older adult (OA) group was significantly older and had lower functional balance, as demonstrated by lower miniBest scores.

**Table 1:** Participants demographics.

	Young adults (*n* = 10) mean (standard deviation)	Older adults (*n* = 11) mean (standard deviation)
Age	21.90 (1.91)	72.64 (5.63)*
Sex (F/M)	4/6	5/6
RBANS	97.00 (10.53)	104.64 (9.89)
Visuospatial/Constructional	87.80 (15.54)	80.09 (7.11)
Attention	107.70 (15.56)	118.36 (15.76)
FES-I	18.20 (1.75)	19.00 (2.49)
MiniBEST	26.89 (0.93)	24.36 (2.25)*

RBANS, repeatable battery for the assessment of neuropsychological status; FES-I, falls efficacy scale-international; *, statistically difference between groups, *p* < 0.05.

### 3.2. Postural control performance

#### 3.2.1. SOT equilibrium score

To achieve residual normality in LMMs, the SOT equilibrium score (Eq) went through outlier removal and log transformation of the data. As the raw data was negatively skewed, the transformed data negatively correlates with the original score. A significant condition effect (*p* < 0.01) was found. Specifically, in comparison to the eyes open condition (estimate: 1.48, standard error: 0.16), eyes closed condition (*b* = 0.53, *p* < 0.01), eyes open sway surrounding condition (*b* = 0.77, *p* < 0.01), eyes open sway platform condition (*b* = 1.44, *p* < 0.01), eyes closed sway platform condition (*b* = 2.02, *p* < 0.01), eyes open sway surrounding and platform condition (*b* = 2.03, *p* < 0.01) all demonstrated higher log transformed equilibrium score, corresponding to higher postural sway. There were no statistically significant age or age condition interaction effects on equilibrium score.

#### 3.2.2. MCT average latency

To achieve model residual normality, average latency went through outlier removal and square-root data transformation. As the raw data was positively skewed, transformed data is in a positive relationship with original data. Significant condition (*p* < 0.01) and age effects (*p* < 0.01) were found for the average latency, but no age × condition interaction effect was found. For age effects, compared to YA (estimate: 11.85, standard error: 0.15), OA demonstrated significantly higher average latencies (*b* = 0.73, *p* < 0.01). For the condition effect, compared to small forward perturbations (estimate: 12.59, standard error: 0.15), forward large perturbations (*b* = −0.42, *p* < 0.05), backward median perturbations (*b* = −0.50, *p* < 0.01), and backward large perturbations (*b* = −0.94, *p* < 0.01) demonstrated statistically significant shorter average latencies.

#### 3.2.3. ADT sway energy

Sway energy score went through outlier removal and achieved model residual normality. Linear mixed effect models indicated significant condition (*p* < 0.01) and age effects (*p* < 0.01) on sway energy scores. For age effects, compared to young adults (estimate: 56.52, standard error: 4.04), older adults demonstrated higher sway energy scores (*b* = 17.34, *p* < 0.01). For condition effect, compared to toe down condition (estimate: 56.36, standard error: 4.04), toe up condition demonstrated higher sway energy score (*b* = 17.50, *p* < 0.01). There were no statistically significant age × condition interaction effects found in any of the measures.

### 3.3. Cortical activities in response to perturbations

In data pre-processing, an average of 0.20 channels (range 0–1) were visually rejected in the YA group and an average of 0.55 channels (range 0–1) were visually rejected in the OA group. For SOT, an average of 0.10 trials (range 0–6) were rejected in the YA group and an average of 0.18 trials (range 0–6) were rejected in the OA group. For MCT, an average of 0.10 trials (range 0–6) were rejected in the YA group, and an average of 0.09 trials (range 0–6) were rejected in the OA group. For ADT, 0 trials were rejected in the YA group and an average of 0.20 trials (range 0–2) were rejected in the OA group.

#### 3.3.1. Cortical activation distribution pattern and ICC results

**Figure 1:**
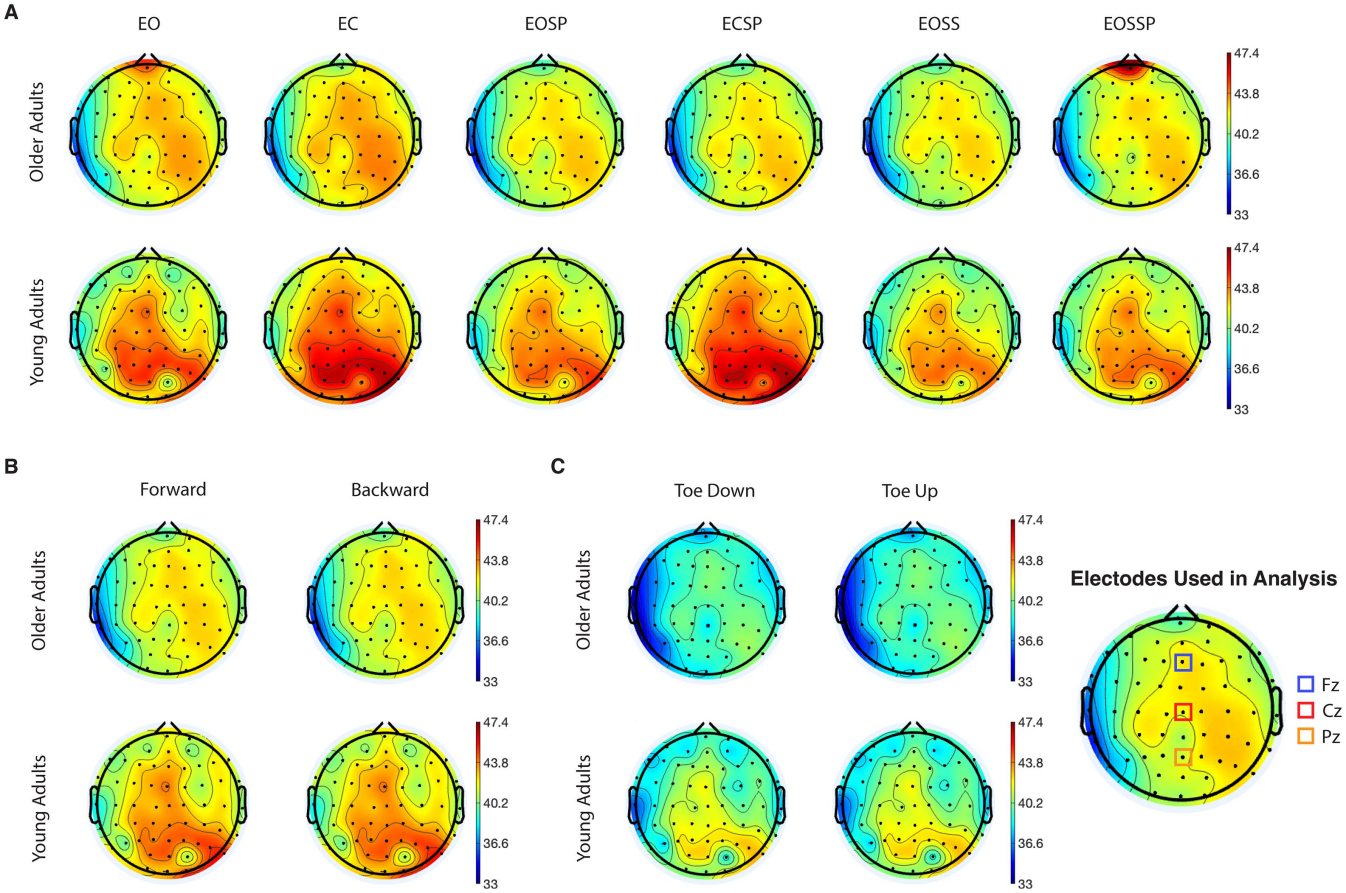
Grand average topographical maps of electroencephalography (EEG) absolute power for beta frequency band during **(A)** sensory organization test, time duration 20s, **(B)** motor control test, time duration 2.5 s, and **(C)** adaptation test, time duration 2.5 s. The red regions correspond to high concentration of maximal (58.5 dB) and blue areas correspond to high concentration of minimal (33 dB). EO, eyes open condition; EC, eyes closed condition; EOSS, eyes open sway surrounding condition; EOSP eyes open sway platform condition; ECSP, eyes closed sway platform condition; EOSSP, eyes open sway surrounding and platform condition.

Figure 1 demonstrates EEG topographic maps for beta-band absolute power separated based on age groups, tests, and conditions. Differences between OA and YA groups can be found in all tests and conditions. Specifically, higher activations in beta-band are observed in the areas around central and right parietal-occipital regions. To access the intertrial reliability of EEG findings, the intraclass correlation value on EEG data before grand averages were performed in SOT, MCT, and ADT tests was calculated. Specifically, the SOT demonstrated a good reliability in all conditions (ICC: 0.875, 95% CI: 0.851–0.895). However, there was a poor reliability in the ADT (ICC: 0.423, 95% CI: 0.338–0.514) and MCT (ICC: 0.416, 95% CI: 0.35–0.481).

#### 3.3.2. SOT relative beta power

**Figure 2:**
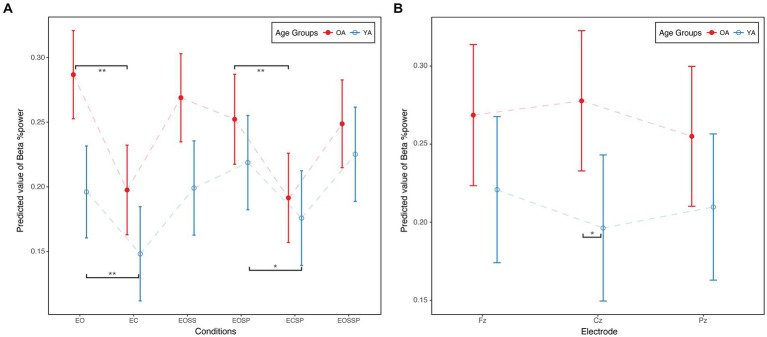
**(A)** Age × condition interaction in SOT. **(B)** Age × electrode interaction in MCT. Beta %Power, beta band relative power; EO, eyes open condition; EC, eyes closed condition; EOSS, eyes open sway surrounding condition; EOSP eyes open sway platform condition; ECSP, eyes closed sway platform condition; EOSSP, eyes open sway surrounding and platform condition. OA, older adults; YA, young adults. *p < 0.05.**p < 0.01.

Linear mixed effect models suggested significant condition (*p* < 0.01), age (*p* < 0.05), electrode (*p* < 0.01), and age condition interaction effects (Figures 2A, *P* < 0.01) on relative beta power. For age effect, compared to YA, OA demonstrated higher relative beta power (*b* = 0.09, *p* < 0.01). For electrode effect, compared to Cz, relative beta power was significantly lower at Pz (*b* = −0.02, *p* < 0.01). For condition effect, compared to the eyes open condition, eye closed condition (*b* = −0.09, *p* < 0.01), eye open sway platform condition (*b* = −0.03, *p* < 0.01), eye close sway platform condition (*b* = −0.10, *p* < 0.01), and eye open sway surrounding and platform condition (*b* = −0.04, *p* < 0.01) demonstrated statistically significant lower relative beta power.

Figure 2A illustrates the age condition interaction effect on relative beta power. Young adults increased relative beta power from the first condition to the last condition, while older adults decreased relative beta power. Moreover, compared to eye open condition, young adults (*b* = 0.048, *p* < 0.01) and older adults (*b* = 0.089, *p* < 0.01) demonstrated lower relative beta power in eye close condition. Similarly, compared to eye open sway platform condition, young adults (b = 0.043, *p* < 0.05) and older adults (*b* = 0.061, *p* < 0.01) demonstrated lower relative beta power in eye close sway platform condition.

#### 3.3.3. MCT relative beta power

Linear mixed effect models suggested significant condition (*p* < 0.01) and age electrode interaction effects (Figures 2B, *P* < 0.05) on relative beta power. Specifically, compared to the forward median amplitude condition, the backward median amplitude condition demonstrated significantly lower relative beta power (*b* = −0.040, *p* < 0.01). For the age electrode interaction, older adults demonstrated statistically significantly higher relative beta power at Cz compared to young adults (*b* = 0.081, *p* < 0.05, Figure 2B).

#### 3.3.4. ADT relative beta power

Linear mixed effect models suggested a significant condition effect (*p* < 0.01) on relative beta power. Specifically, compared to the toe up condition, the toe down condition demonstrated significantly higher relative beta power (*b* = 0.03, *p* < 0.01).

### 3.4. Correlation between cortical activation and postural control

For relative beta power, only a very weak negative correlation was found in overall level at Pz ( ρ=−0.160 , *p* < 0.10). The age subgroup analysis revealed no correlation in OA, but a significant moderate negative correlation in YA at Pz ( ρ=−0.436 , *p* < 0.001). Moreover, the age and condition subgroup analysis at Pz suggested that in the eyes open and eyes open sway surrounding conditions, higher relative beta power was strongly correlated with lower equilibrium scores in YA (EO: ρ=−0.81 , *p* < 0.01; EOSS: ρ=−0.69 , *p* < 0.05). No other statistically significant correlation was identified in subgroup analysis.

Significant correlations were found between relative beta power and average latency score at Fz ( ρ=0.214 , *p* < 0.05) and Cz ( ρ=0.300 , *p* < 0.01) during MCT paradigms. Moreover, subgroup analysis found positive correlations between relative beta power and average latency score in OA at Fz ( ρ=0.299 , *p* < 0.05) and Cz ( ρ=0.315 , *p* < 0.05), but not in YA. Moreover, the age and condition subgroup analysis at Cz suggested that in the forward small perturbation (FS) condition, higher relative beta power was strongly correlated with higher average latency in OA ( ρ=0.7 , *p* < 0.05). No statistically significant relationship was detected between relative beta power and sway energy during ADT paradigms.

## 4. Discussion

This study investigated the effects of aging on cortical activities in response to sensory manipulation and different types of mechanical perturbation while standing and their relationships with condition-specific postural control performance and function balance ability. Our main findings were that: (1) under sensory manipulation, OA demonstrate significantly higher beta power at all postural control-related cortical areas; (2) under rapid mechanical perturbation, OA demonstrate significantly higher relative beta power at central areas; (3) As task difficulty increased, YA increased relative beta power while OA demonstrated decreased relative beta power; (4) during sensory manipulation with mild mechanical perturbations, specifically in the easier eyes open conditions, higher relative beta power at the parietal area in YA was associated with worse postural control performance; and (5) under rapid mechanical perturbation, specifically in novel conditions, higher relative beta power at the central area in OA are associated with longer movement latency. However, poor reliability measures of cortical activity assessments were found during MCT and ADT, which limits the ability to interpret the reported results.

Confirming our first hypothesis, older adults displayed significantly higher relative beta power at postural control-related cortical areas, relative to younger adults. These observations were consistent with the literature, where higher cortical engagement has been found in older adults under challenging postural conditions (Seidler et al., 2010). Moreover, our results suggest that the compensatory cortical activity seen in older adults is task specific, meaning that aging influences cortical oscillatory activity differently depending on the type of postural perturbation. Our results further support the compensation theory in aging functional brain recruitment patterns (Seidler et al., 2010). Compensation theory suggests that older adults require additional brain activity to perform the task at the same level as young adults, as was observed in the SOT paradigms.

Confirming our second hypothesis, there were significant age by condition interaction effects in relative beta power in the sensory organization test. As task difficulty increased, greater beta band relative power was found in young adults, consistent with prior work (Ghosn et al., 2020). However, decreased beta band relative power was found in older adults as task difficulty increased. This finding is consistent with recent work in older adults, which found increased beta desynchronization as balance demands increase (Malcolm et al., 2021). Combined with the significantly higher general relative beta power in older adults, it is very likely that older adults already had reached a limit in cortical activity with eyes open and may have been unable to further increase beta activity as balance demands increased. Alternatively, the decrease in beta activity in older adults may be associated with beta desynchronization and use of voluntary-controlled movement strategies (Seeber et al., 2014) to overcome the postural control challenge brought about increased task difficulties, rather than the use of automatic postural responses to the sensory perturbations in young adults.

Consistent with prior work (Ghosn et al., 2020), higher relative beta power was correlated with worse postural control performance under sensory manipulation and rapid mechanical perturbations. Further, postural control-EEG connectivity has been found to result in positive beta oscillatory networks in older adults, such that increased beta network connectivity has been found with increased sway (Ibitoye et al., 2021). As beta power is sensitive to both sensory and mechanical perturbations and aging, cortical beta activity may be a good electrophysiological marker to assess and predict the postural control ability of an individual in the context of aging.

The present study has several limitations. First, the primary outcome measurement was focused on power spectral density, which was calculated on the time windows of each trial of each test condition. Due to this limitation, in mechanical perturbation tests, which include a clear perturbation onset, we cannot investigate the corresponding changes before and after the perturbation onset. Analysis focus on before and after perturbation is needed to investigate whether the changes of beta power is related to anxiety, fear and lack of confidence about the balance task. Second, this project was focused on using well-established clinical instrumented balance tests MCT and ADT to introduce mechanical perturbations, which has limited repeated trials and demonstrated poor reliability in EEG results. Future studies investigating cortical control of balance specific to mechanical perturbation should include larger repeated trials to improve intra-trial reliability. Third, in EEG preprocessing, we implemented a grand average to minimize the trial effect, resulting in limited data points in correlation analysis, which led to a lower correlation coefficient and non-significant relationship in subgroup analysis. Thus, future work should examine a larger sample size to make more generalizable conclusions regarding the relationship between cortical activities and postural control and balance performance. The current study focused on cortical activities and postural control performance, which indicates the shift from an automatic postural response towards a more cortically engaged strategy due to aging. However, evidence from the peripheral motor system, such as muscle activation patterns, is needed to further explain and confirm these observations. Thus, future work should incorporate electromyography and cortical-muscular coherence to connect cortical activities and movement executions.

## Data availability statement

The raw data supporting the conclusions of this article will be made available by the authors, without undue reservation.

## Ethics statement

The studies involving human participants were reviewed and approved by Institutional Review Board of the University of Illinois Urbana Champaign. The patients/participants provided their written informed consent to participate in this study.

## Author contributions

YH, SP, and MH contributed to study design, revised the manuscript. YH and MH carried out participant recruitment, YH carried out the data collection, drafted the manuscript. All authors contributed to the article and approved the submitted version.

## Conflict of interest

The authors declare that the research was conducted in the absence of any commercial or financial relationships that could be construed as a potential conflict of interest.

## Publisher’s note

All claims expressed in this article are solely those of the authors and do not necessarily represent those of their affiliated organizations, or those of the publisher, the editors and the reviewers. Any product that may be evaluated in this article, or claim that may be made by its manufacturer, is not guaranteed or endorsed by the publisher.

## References

[ref1] Adkin A. L. Quant S. Maki B. E. McIlroy W. E. (2006). Cortical responses associated with predictable and unpredictable compensatory balance reactions. Exp. Brain Res. 172, 85–93. doi: 10.1007/s00221-005-0310-9, PMID: 16418848

[ref2] Barollo F. Hassan M. Petersen H. Rigoni I. Ramon C. Gargiulo P.. (2022). Cortical pathways during postural control: new insights from functional EEG source connectivity. IEEE Trans. Neural Syst. Rehabil. Eng. 30, 72–84. doi: 10.1109/TNSRE.2022.3140888, PMID: 34990367

[ref3] Bergen G. Stevens M. R. Burns E. R. (2016). Falls and fall injuries among adults aged ≥65 years — United States, 2014. MMWR Morb. Mortal. Wkly Rep. 65, 993–998. doi: 10.15585/mmwr.mm6537a2, PMID: 27656914

[ref42] Burns E. Kakara R. (2018). Deaths from Falls Among Persons Aged ≥65 Years — United States, 2007–2016. Morb. Mortal. Wkly. Rep. 67:509–514. doi: 10.15585/mmwr.mm6718a1 PMC5944976 29746456

[ref4] Chang C. J. Yang T. F. Yang S. W. Chern J. S. (2016). Cortical modulation of motor control biofeedback among the elderly with high fall risk during a posture perturbation task with augmented reality. Front. Aging Neurosci. 8, 1–13. doi: 10.3389/fnagi.2016.00080, PMID: 27199732 PMC4848299

[ref5] Cook S. E. Marsiske M. McCoy K. J. M. (2009). The use of the modified telephone interview for cognitive status (TICS-M) in the detection of amnestic mild cognitive impairment. J. Geriatr. Psychiatry Neurol. 22, 103–109. doi: 10.1177/0891988708328214, PMID: 19417219 PMC2913129

[ref6] Delbaere K. Close J. C. T. Mikolaizak A. S. Sachdev P. S. Brodaty H. Lord S. R. (2010). The falls efficacy scale international (FES-I). A comprehensive longitudinal validation study. Age Ageing 39, 210–216. doi: 10.1093/AGEING/AFP225, PMID: 20061508

[ref7] Evans J. D. (1996). Straightforward Statistics for the Behavioral Sciences. Pacific Grove, CA: Brooks/Cole Pub. Co.

[ref8] Francis C. A. Franz J. R. O’Connor S. M. Thelen D. G. (2015). Gait variability in healthy old adults is more affected by a visual perturbation than by a cognitive or narrow step placement demand. Gait Posture 42, 380–385. doi: 10.1016/j.gaitpost.2015.07.006, PMID: 26233581 PMC4591170

[ref9] Franz J. R. Francis C. A. Allen M. S. O’Connor S. M. Thelen D. G. (2015). Advanced age brings a greater reliance on visual feedback to maintain balance during walking. Hum. Mov. Sci. 40, 381–392. doi: 10.1016/j.humov.2015.01.012, PMID: 25687664 PMC4372858

[ref10] Franz J. R. Francis C. A. Allen M. S. Thelen D. G. (2017). Visuomotor entrainment and the frequency-dependent response of walking balance to perturbations. IEEE Trans. Neural Syst. Rehabil. Eng. 25, 1135–1142. doi: 10.1109/TNSRE.2016.2603340, PMID: 28113592 PMC5623133

[ref11] Ghosn N. J. Palmer J. A. Borich M. R. Ting L. H. Payne A. M. (2020). Cortical Beta oscillatory activity evoked during reactive balance recovery scales with perturbation difficulty and individual balance ability. Brain Sci. 10:860. doi: 10.3390/brainsci10110860, PMID: 33207570 PMC7697848

[ref12] Goodworth A. Perrone K. Pillsbury M. Yargeau M. (2015). Effects of visual focus and gait speed on walking balance in the frontal plane. Hum. Mov. Sci. 42, 15–26. doi: 10.1016/j.humov.2015.04.004, PMID: 25918828

[ref13] Hartholt K. A. Van Beeck E. F. Polinder S. Van Der Velde N. Van Lieshout E. M. M. Panneman M. J. M.. (2011). Societal consequences of falls in the older population: injuries, healthcare costs, and long-term reduced quality of life. J. Trauma—Injury, Inf. Crit. Care 71, 748–753. doi: 10.1097/TA.0b013e3181f6f5e5, PMID: 21045738

[ref14] Honaker J. A. Criter R. E. (2013). “Testing, posturography” in Encyclopedia of Otolaryngology, Head and Neck Surgery (Berlin Heidelberg: Springer), 2757–2765.

[ref15] Ibitoye R. T. Castro P. Desowska A. Cooke J. Edwards A. E. Guven O.. (2021). Small vessel disease disrupts EEG postural brain networks in ‘unexplained dizziness in the elderly’. Clin. Neurophysiol. 132, 2751–2762. doi: 10.1016/J.CLINPH.2021.07.027, PMID: 34583117 PMC8559782

[ref16] Jacobs J. V. Fujiwara K. Tomita H. Furune N. Kunita K. Horak F. B. (2008). Changes in the activity of the cerebral cortex relate to postural response modification when warned of a perturbation. Clin. Neurophysiol. 119, 1431–1442. doi: 10.1016/j.clinph.2008.02.015, PMID: 18397840 PMC2443739

[ref17] Jacobs J. V. Horak F. B. (2007). Cortical control of postural responses. J. Neural Transm. 114, 1339–1348. doi: 10.1007/s00702-007-0657-0, PMID: 17393068 PMC4382099

[ref18] Johansson J. Nordström A. Gustafson Y. Westling G. Nordström P. (2017). Increased postural sway during quiet stance as a risk factor for prospective falls in community-dwelling elderly individuals. Age Ageing 46, 964–970. doi: 10.1093/ageing/afx083, PMID: 28531243

[ref19] Kanekar N. Aruin A. S. (2014). The effect of aging on anticipatory postural control. Exp. Brain Res. 232, 1127–1136. doi: 10.1007/s00221-014-3822-3, PMID: 24449006 PMC3954907

[ref20] Kannus P. Parkkari J. Koskinen S. Niemi S. Palvanen M. Järvinen M.. (1999). Fall-induced injuries and deaths among older adults. J. Am. Med. Assoc. 281, 1895–1899. doi: 10.1001/jama.281.20.1895, PMID: 10349892

[ref21] Lord S. R. Delbaere K. Sturnieks D. L. (2018). Aging. Handb. Clin. Neurol., 159:157–171. doi: 10.1016/B978-0-444-63916-5.00010-0 30482312

[ref22] Maki B. E. McIlroy W. E. (2007). Cognitive demands and cortical control of human balance-recovery reactions. J. Neural Transm. 114, 1279–1296. doi: 10.1007/s00702-007-0764-y, PMID: 17557125

[ref23] Malcolm B. R. Foxe J. J. Joshi S. Verghese J. Mahoney J. R. Molholm S.. (2021). Aging-related changes in cortical mechanisms supporting postural control during base of support and optic flow manipulations. Eur. J. Neurosci. 54, 8139–8157. doi: 10.1111/EJN.15004, PMID: 33047390

[ref24] Mcguirk T. E. (2005). The use of computerized dynamic posturography to assess the balance in individuals with Parkinson’ s disease [Master’s thesis, Virginia Commonwealth University]. doi: 10.25772/BT6M-8571

[ref25] Mochizuki G. Sibley K. M. Cheung H. J. Camilleri J. M. McIlroy W. E. (2009). Generalizability of perturbation-evoked cortical potentials: Independence from sensory, motor and overall postural state. Neurosci. Lett. 451, 40–44. doi: 10.1016/j.neulet.2008.12.020, PMID: 19110034

[ref26] NeuroCom International (2008). Balance manager systems clinical interpretation guide, computerized dynamic posturography. Clackamas: NeuroCom Internation.

[ref27] O’Connor S. M. Kuo A. D. (2009). Direction-dependent control of balance during walking and standing. J. Neurophysiol. 102, 1411–1419. doi: 10.1152/jn.00131.2009, PMID: 19553493 PMC2746770

[ref28] O’Connor S. M. Xu H. Z. Kuo A. D. (2012). Energetic cost of walking with increased step variability. Gait Posture 36, 102–107. doi: 10.1016/j.gaitpost.2012.01.014, PMID: 22459093 PMC3372656

[ref29] O’loughlin J. L. Robitaille Y. Boivin J. F. Suissa S. (1993). Incidence of and risk factors for falls and injurious falls among the community-dwelling elderly. Am. J. Epidemiol. 137, 342–354. doi: 10.1093/oxfordjournals.aje.a116681 8452142

[ref30] Papegaaij S. Taube W. Baudry S. Otten E. Hortobágyi T. (2014). Aging causes a reorganization of cortical and spinal control of posture. Front. Aging Neurosci. 6, 1–15. doi: 10.3389/fnagi.2014.00028, PMID: 24624082 PMC3939445

[ref31] Pollock A. S. Durward B. R. Rowe P. J. Paul J. P. (2000). What is balance? Clin. Rehabil. 14, 402–406. doi: 10.1191/0269215500cr342oa 10945424

[ref32] Quijoux F. Vienne-Jumeau A. Bertin-Hugault F. Zawieja P. Lefèvre M. Vidal P.-P.. (2020). Center of pressure displacement characteristics differentiate fall risk in older people: a systematic review with meta-analysis. Ageing Res. Rev. 62:101117. doi: 10.1016/j.arr.2020.101117, PMID: 32565327

[ref33] Randolph C. Tierney M. C. Mohr E. Chase T. N. (1998). The repeatable battery for the assessment of neuropsychological status (RBANS): preliminary clinical validity. J. Clin. Exp. Neuropsychol. 20, 310–319. doi: 10.1076/jcen.20.3.310.823, PMID: 9845158

[ref35] Roman-Liu D. (2018). Age-related changes in the range and velocity of postural sway. Arch. Gerontol. Geriatr. 77, 68–80. doi: 10.1016/j.archger.2018.04.007, PMID: 29684741

[ref36] Rubega M. Formaggio E. di Marco R. Bertuccelli M. Tortora S. Menegatti E.. (2021). Cortical correlates in upright dynamic and static balance in the elderly. Sci. Rep. 11, 14132–14115. doi: 10.1038/s41598-021-93556-3, PMID: 34238987 PMC8266885

[ref37] Seeber M. Scherer R. Wagner J. Solis-Escalante T. Müller-Putz G. R. (2014). EEG beta suppression and low gamma modulation are different elements of human upright walking. Front. Hum. Neurosci. 8, 1–9. doi: 10.3389/fnhum.2014.00485, PMID: 25071515 PMC4086296

[ref38] Seidler R. D. Bernard J. A. Burutolu T. B. Fling B. W. Gordon M. T. Gwin J. T.. (2010). Motor control and aging: links to age-related brain structural, functional, and biochemical effects. Neurosci. Biobehav. Rev. 34, 721–733. doi: 10.1016/j.neubiorev.2009.10.005, PMID: 19850077 PMC2838968

[ref39] Shepard N. T. Janky K. (2008). “Background and technique of computerized dynamic posturography” in Balance Function Assessment and Management. eds. Jacobson G. P. Shepard N. T. Stach B. (San Diego: Plural Publishing)

[ref40] Smith B. A. Jacobs J. V. Horak F. B. (2014). Effects of amplitude cueing on postural responses and preparatory cortical activity of people with Parkinson disease. J. Neurol. Phys. Ther. 38, 207–215. doi: 10.1097/NPT.0000000000000058, PMID: 25198870 PMC4322905

[ref41] St George R. J. Hinder M. R. Puri R. Walker E. Callisaya M. L. (2021). Functional near-infrared spectroscopy reveals the compensatory potential of pre-frontal cortical activity for standing balance in young and older adults. Neuroscience 452, 208–218. doi: 10.1016/j.neuroscience.2020.10.027, PMID: 33197501

[ref43] Sullivan E. V. Rose J. Rohlfing T. Pfefferbaum A. (2009). Postural sway reduction in aging men and women: relation to brain structure, cognitive status, and stabilizing factors. Neurobiol. Aging 30, 793–807. doi: 10.1016/j.neurobiolaging.2007.08.021, PMID: 17920729 PMC2684797

[ref44] Trueblood P. R. Rivera M. Lopez C. Bentley C. Wubenhorst N. (2018). Age-based normative data for a computerized dynamic posturography system that uses a virtual visual surround environment. Acta Otolaryngol. 138, 597–602. doi: 10.1080/00016489.2018.1429653, PMID: 29390922

[ref45] Tsai Y. Y. Chen Y. C. Zhao C. G. Hwang I. S. (2022). Adaptations of postural sway dynamics and cortical response to unstable stance with stroboscopic vision in older adults. Front. Physiol. 13:1659. doi: 10.3389/FPHYS.2022.919184/BIBTEX PMC9465385 36105297

[ref46] Tse Y. Y. F. Petrofsky J. S. Berk L. Daher N. Lohman E. Laymon M. S.. (2013). Postural sway and rhythmic electroencephalography analysis of cortical activation during eight balance training tasks. Med. Sci. Monit. 19, 175–186. doi: 10.12659/MSM.883824, PMID: 23470794 PMC3628716

[ref47] Vanicek N. King S. A. Gohil R. Chetter I. C. Coughlin P. A. (2013). Computerized dynamic Posturography for postural control assessment in patients with intermittent claudication. J. Vis. Exp.:e51077. doi: 10.3791/51077, PMID: 24378378 PMC4047968

[ref48] Wittenberg E. Thompson J. Nam C. S. Franz J. R. (2017). Neuroimaging of human balance control: a systematic review. Front. Hum. Neurosci. 11, 1–25. doi: 10.3389/fnhum.2017.00170, PMID: 28443007 PMC5385364

