# Supplementary material for: Beta cortical oscillatory activities and their relationship to postural control in a standing balance demanding test: influence of aging
Source: Front Aging Neurosci. 2023 May 5;15:1126002. doi: 10.3389/fnagi.2023.1126002 (PMC10196243; doi:10.3389/fnagi.2023.1126002)
Supplement: Supplementary file 1 [file Data_Sheet_1.DOCX]

Supplementary Material

# Supplementary Method

## EEG Raw Data Preprocessing

Raw EEG data was imported into EEGLAB (version 2020.0) using MATLAB (The MathWorks, Natick, MA, USA) for preprocessing. Data was high-passed filtered at 1Hz to remove drift and Low-pass filtered at 55Hz to remove line noise. A band-specific filter for 60Hz was applied due to the environment line noise in the lab. EEG artifacts associated with eye and other muscle movements was removed using independent component analysis (ICA). Based on the topography, spectra, and trial-to-trial characteristics of ICA components, good fit ICA components were selected and used to generate back-projected EEG data, which is referred to as clean EEG.

## Statistical Analysis

For primary outcome measurements, outliers were detected and removed using the Interquartile Range (IQR) method with a 1.5 IQR cut off. To achieve residual normality, log or square-root data transformation were performed based on the direction and level of skewness of the data set. Linear mixed effect models (LMMs) were used to identify the cohort differences for cortical activities and postural control performance. Age group and Test conditions were used as fixed effects, and subject ID was used as the random effect to construct the LMMs for primary outcomes. To test the significance of the fixed effects, different models were constructed to include a different set of fixed effects and their interactions. After the models were constructed, the likelihood ratio test (LRT), which can achieve by the ‘ANOVA’ function in ‘lme4’ package in R, was performed to test the significance of fixed effects and their interactions (reference: LME tutorial). For each of the models, a QQ plot of residual of the model was used to test the normality of residual distribution, and a residual versus fits plot was used to identify non-linearity, unequal error variances, or outliers. Moreover, the best fitting linear mixed model was selected based on the Akaike information criterion (AIC) and Bayesian information criterion (BIC) estimated from the LRT. When e significant interaction effects were found, Least Square Means (LSM) posthoc comparisons were performed, and a p-value <0.05 was considered statistically significant.
